# Supplementary material for: Joint dimension reduction and clustering analysis of single-cell RNA-seq and spatial transcriptomics data
Source: Nucleic Acids Res. 2022 Mar 29;50(12):e72. doi: 10.1093/nar/gkac219 (PMC9262606; doi:10.1093/nar/gkac219)
Supplement: gkac219_Supplemental_Files [file gkac219_supplemental_files.zip › supp_Tab_DR-SC_R1.pdf]

Supplementary Figures to
Joint dimension reduction and clustering analysis for single-cell RNA-seq and spatial transcriptomics data

Wei Liu[1,2], Xu Liao[2], Yi Yang[2], Huazhen Lin[3], Joe Yeong[4], Xiang Zhou[5*], Xingjie Shi[1,6*], Jin Liu[2*]

[1] Academy of Statistics and Interdisciplinary Sciences, East China Normal University, Shanghai, 200062, China

[2] Centre for Quantitative Medicine, Health Services & Systems Research, Duke-NUS Medical School, Singapore 169857, Singapore

[3]Center of Statistical Research and School of Statistics, Southwestern University of Finance and Economics, Chengdu, 611130, China

[4]Institute of Molecular and Cell Biology(IMCB), Agency of Science, Technology and Research(A*STAR), Singapore, 138673, Singapore

[5] Department of Biostatistics, University of Michigan

[6]Key Laboratory of Advanced Theory and Application in Statistics and Data Science-MOE, School of Statistics, East China Normal University, Shanghai, 200062, China

Contents

1 Captions for Supplementary Tables **1**

1 Captions for Supplementary Tables

- Table S1. Mean and covariance structures for both Simulation 1 and Simulation 2.

*To whom correspondence should be addressed. Jin Liu (jin.liu@duke-nus.edu.sg), Xingjie Shi(xjshi@fem.ecnu.edu.cn), and Xiang Zhou (xzhousph@umich.edu)

- Table S2. Summary information for all 12 DLPFC samples in LIBD data.
- Table S3. Data information for 16 scRNAseq datasets used for benchmarking dimension reduction methods through trajectory inference.
- Table S4. Details of all identified differentially expressed genes across nine cell types.
- Table S5. Summary of all spatial transcriptomics datasets used in the analysis.
- Table S6. Details of all identified spatially variable genes among all 12 DLPFC samples in LIBD data.
- Table S7. Top five pathways identified for each category among all 12 DLPFC samples in LIBD data.
- Table S8. Details of all identified spatially variable genes in mouse olfactory bulb data.
- Table S9. Details of all identified differentially expressed genes in brain regions of mouse embryo data.
